# A tool for determining duration of mortality events in archaeological assemblages using extant ungulate microwear

**DOI:** 10.1038/srep17330

**Published:** 2015-11-30

**Authors:** Florent Rivals, Luce Prignano, Gina M. Semprebon, Sergi Lozano

**Affiliations:** 1 Institució Catalana de Recerca i Estudis Avançats (ICREA), Barcelona, Spain; 2 Institut Català de Paleoecologia Humana i Evolució Social (IPHES), C. Marcellí Domingo s/n, Campus Sescelades URV (Edifici W3), 43007 Tarragona, Spain; 3 Universitat Rovira i Virgili (URV), Area de Prehistoria, Avinguda de Catalunya 35, 43002 Tarragona, Spain; 4 Departament de Física Fonamental, Universitat de Barcelona, Facultat de Física-C. Martí i Franqués 1, 08028 Barcelona, Spain; 5 Bay Path University, 588 Longmeadow Street, Longmeadow, MA 01106, USA

## Abstract

The seasonality of human occupations in archaeological sites is highly significant for the study of hominin behavioural ecology, in particular the hunting strategies for their main prey-ungulates. We propose a new tool to quantify such seasonality from tooth microwear patterns in a dataset of ten large samples of extant ungulates resulting from well-known mass mortality events. The tool is based on the combination of two measures of variability of scratch density, namely standard deviation and coefficient of variation. The integration of these two measurements of variability permits the classification of each case into one of the following three categories: (1) short events, (2) long-continued event and (3) two separated short events. The tool is tested on a selection of eleven fossil samples from five Palaeolithic localities in Western Europe which show a consistent classification in the three categories. The tool proposed here opens new doors to investigate seasonal patterns of ungulate accumulations in archaeological sites using non-destructive sampling.

The study of mortality events of large game at prehistoric archaeological sites provides significant information for the understanding of ecological management in early phases of human evolution. Among other aspects, researchers usually focus on the approximate duration of these events and their seasonality. Seasonality of the events of large game procurement in archaeological sites is often studied through the analysis of (1) the patterns of tooth eruption, wear and replacement^1^,^2^,^3^,^4^, (2) cementochronology^5^,^6^,^7^, or (3) stable isotope analysis of serial samples^8^,^9^. However these methods lack precision or have restrictive requirements. The first method requires large numbers of young individuals and does not take into account adults, whereas the other two methods are destructive. This makes the duration of the mortality events a parameter difficult to estimate, especially in archaeological sites.

An alternative to the above-mentioned methods is to use tooth microwear as a proxy for estimating the relative duration of these events. Tooth microwear, which analyses the microscopic features produced by food items on the surface of teeth, is known to reflect seasonal shifts in diets in ungulates^10^,^11^,^12^. Tooth microwear has been used in the past four decades to provide information about the dietary traits of extant and extinct mammals such as ungulates and primates, among others^13^,^14^,^15^,^16^,^17^,^18^,^19^. Many such studies using different methods of observation and analysis have reported a correlation between tooth microwear patterns and the dietary traits of extant species. For example, in ungulates, grazers tend to have significantly higher numbers of scratches than leaf browsers^14^,^17^,^20^. Each of these microwear features record unique chewing events and the food items associated with them. Consequently, the microwear patterns observed at the surface of teeth are constantly worn away and overprinted by new features as the tooth wears down. This results in a high turnover of the microwear patterns which reflects the diet of an individual recorded during a short time, such as days or weeks^21^,^22^,^23^. This phenomenon is known as the “Last Supper Effect”^21^. Consequently, teeth sampled at different times display microwear patterns related to each time frame, and will reflect seasonal changes in the diet^11^,^12^,^24^,^25^.

Nevertheless, under certain circumstances, the “Last Supper Effect” might potentially bias the dietary interpretation of samples from fossil assemblages^26^. For example, in the case of natural mortality, fossil assemblages may accumulate over different periods of time: e.g., over long periods of attritive mortality producing assemblages of animals dead during a single year or more, seasonal accumulations (drought or winter mortality), or very short events of catastrophic mortality (e.g. volcanic eruption). This can be observed in archaeological sites where large mammal assemblages result from the accumulation of prey by hominids during events of various durations: i.e., short-term occupations, seasonal occupations or longer occupations (several seasons or longer). If an assemblage is the result of a seasonal or even a shorter mortality event, tooth microwear will only represent a snapshot of the diet of a species at that time. However, the high turnover propensity of microwear features is often an essential asset to study more typical seasonal changes in diets, especially when the depositional event that created an assemblage is short.

Building on these particularities of the “Last Supper Effect”, this paper provides insights on how to take advantage of microwear analysis of modern tooth samples to better assess the duration of mortality events in archaeological assemblages. The first objective of the paper is to test the use of variability measurements in microwear patterns as a proxy to assess the duration of the mortality events in different types of samples of ungulate teeth. We sampled and analysed tooth surfaces from extant ungulate assemblages resulting from mass mortality events and from large samples with excellent known data for each individual. The second objective is to test this application on specific cases from archaeological assemblages where previous archaeological studies defined the context of human occupations. To this end, we selected five Middle and Upper Pleistocene sites in Europe (Spain, France, and Germany) with a good record of archaeological remains and where microwear data were available.

Mass mortality samples of large mammals are numerous and well-documented through many publications and reports which list the following causes for such mortality: extreme winters, droughts, starvation, violent storms, volcanic eruptions, quagmires, or natural fires^27^,^28^,^29^,^30^,^31^,^32^,^33^,^34^,^35^. Unfortunately, most of the animals that died during these mass mortality events were studied in the field but not collected and conserved.

The material available in museums and collections usually consists of samples coming from various populations and different geographic areas, most of the time without very precise information available. Conversely, in this study, we analysed large samples of animals (1) coming from single populations (i.e. from a very restricted geographic area) or (2) resulting from a catastrophic death (i.e. massive and sudden) with precise records of their date of death. Such samples are unique, and thus very rare to find. We have, however, located ten samples which fulfil these criteria and with mortality durations spanning from one day to a several years. The samples span a wide diversity of taxonomic groups including Cervidae (*Cervus elaphus*, *Cervus nippon*, *Odocoileus hemionus*, *Rangifer tarandus*), Antilocapridae (*Antilocapra americana*), Camelidae (*Lama guanicoe*), and Equidae (*Equus quagga*) as well as various geographical origins in Europe, Africa, Asia, North America and South America. The locations where these data were collected from are shown in Fig. 1 (numerical identifiers).

We sampled fossil teeth of ungulates from archaeological sites in Europe ranging in age from the middle to late Pleistocene. The species sampled belong to the same families as those from the extant dataset: Cervidae (*Cervus elaphus* and *Rangifer tarandus*), Bovidae (*Bison priscus* and *Bos primigenius*), and Equidae (*Equus ferus*). The localities and species were selected following strict criteria: good stratigraphic control (sites with modern excavations which are well dated), well preserved specimens (i.e., those without weathering or chemical alteration), and those with large numbers of specimens available. The locations of the five archaeological sites are presented in Fig. 1 (alphabetical identifiers).

A comprehensive summary of all the samples is provided in the *Methods* section and the Supplementary Information.

## Results

Once these samples were collected, we quantitatively examined the tooth microwear patterns. Following the protocol proposed by Solounias and Semprebon^14^ and Semprebon *et al.*^36^, we obtained the density of scratches per standard area of 0.16 mm^2^ on the occlusal surface of the upper or lower second molar. See the *Methods* section for a complete description of the analytical protocol.

The resulting dataset contains one value of density of scratches for each individual. Then, in order to address our first goal (i.e. relating the duration of a mortality event with the variability of microwear patterns among deceased individuals), we started by characterising the variability of the density of scratches in our dataset.

**Figure 2:**
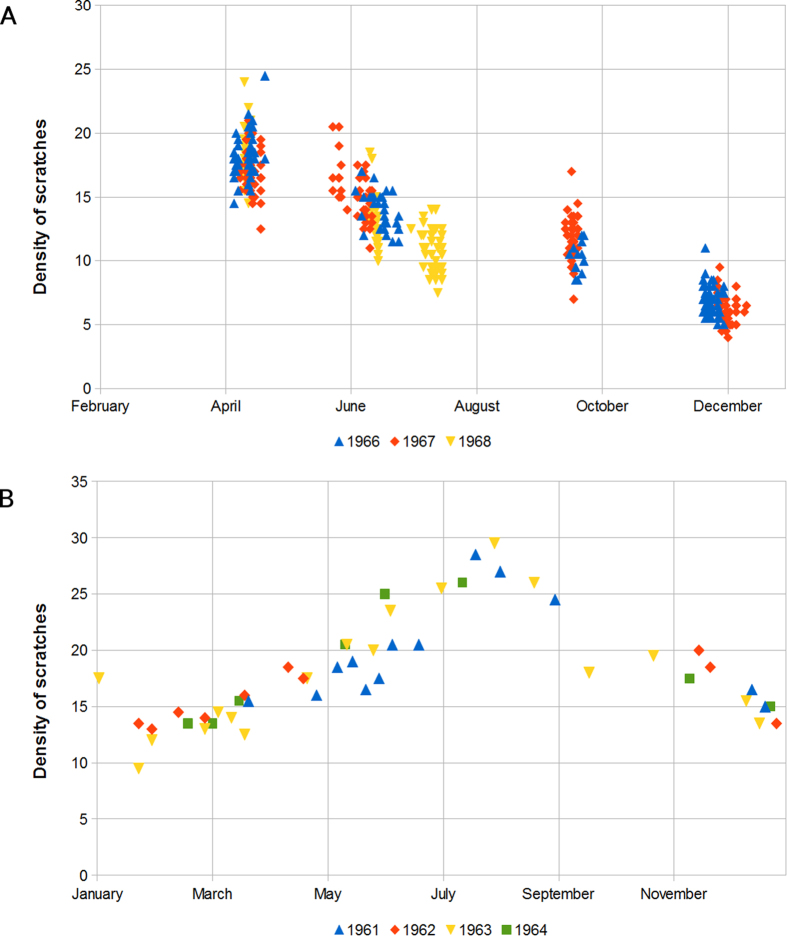
Density of scratches as a function of the date of death (month and day) from the datasets #1 (Panel A) and #4 (Panel B). Each data point stands for an individual. Vertically aligned points correspond to individual variability within the same mortality event. The variability range changes across seasons and colour coding highlights that the seasonal pattern is independent of the year.

At first sight, one can observe that the density of scratches varies from individual to individual (even if they correspond to the same mortality event). For example, in the case of the caribou from Canada [sample #1] there is variability among animals that died on the same day (Fig. 2A). In all the analysed cases, the raw data also show this variability (Datasets are available from the Dryad Digital Repository: http://dx.doi.org/10.5061/dryad.9fp8k). Such an individual heterogeneity, which is related to characteristics such as gender, age or healthiness, can be regarded as a first source of variability. Additionally, an exploratory analysis of the results from the cases which data are available for several seasons (i.e. *Rangifer tarandus* [sample #1] and *Odocoileus hemionus* [sample #4]) revealed that the average density of scratches depended on the season within the year when the deaths occurred (Fig. 2). Specifically, independently of the species or habitat considered, the average density reached the highest values in spring (e.g. for *Rangifer tarandus*) or summer (e.g. for *Odocoileus hemionus*) and the lowest ones in fall/winter, which corresponds to an increase of the consumption of abrasive vegetation (like grass) in spring or summer for the two populations considered. Consequently, we identified two main sources of variability in the density of scratches, namely differences among individuals and season-related changes in environmental conditions.

We plotted and visually inspected the distribution of scratch densities of all the samples. When high resolution data were available, we also subdivided samples corresponding to long duration (e.g. a year) into shorter ones (e.g. a season, a month or a day) and compared their distributions. Interestingly, we found that the influence of these two sources of variability gets more or less relevant depending on the temporal window we are considering when analysing the data.

1. If the time window was a season or shorter, we mainly saw heterogeneity due to individual characteristics.
2. If the time window was longer than a season, season-dependent variability played a major role, and the overall dispersion increased significantly.

This relationship between variability source and temporal window is represented on Fig. 2 for datasets #1 and #4.

Taking this into account, our next goal was to exploit the above-mentioned associations to easily determine time windows of mortality events from data on scratch densities. In order to do this, we needed to quantitatively assess variability.

### Classification applying usual measures of variability

As a first approach, we applied two common measures of variability, namely the Standard Deviation (SD) and Coefficient of Variation (CV). Specifically, we calculated both magnitudes for a selection of the modern samples, and compared the resulting values with the corresponding duration. Such a comparative exercise revealed that neither the SD nor the CV consistently distinguished short from long-term death events if included samples represented different seasons.

**Figure 3:**
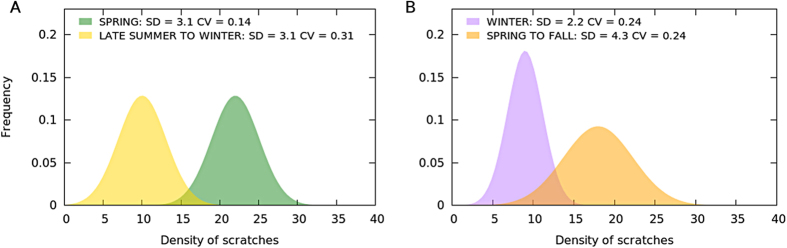
Frequency distribution of the density of scratches in individuals that died in different mortality events. Both standard deviation (SD) and coefficient of variation (CV) have problems distinguishing mortality time windows in certain cases. Panel (**A)** (left): Hypothetical scenario where SD cannot distinguish a single event in a warm season from a longer one in a colder part of the year. Panel (**B)** (right): Hypothetical scenario where CV cannot distinguish a single event in a cold season from a longer one in a warm part of the year.

SD alone may not be able to properly distinguish death events concentrated in the warm season from those distributed during a larger time interval. In the latter case, however, the average number of marks will be smaller (See Fig. 3A). This would increase CV, making it possible to discriminate between the two scenarios. On the contrary, considering CV alone makes it difficult to separate short mortality events concentrated in the colder seasons from events distributed on a longer time interval. These two cases are easily distinguishable using SD, which is low in the former and high for the latter (See Fig. 3B).

**Figure 4:**
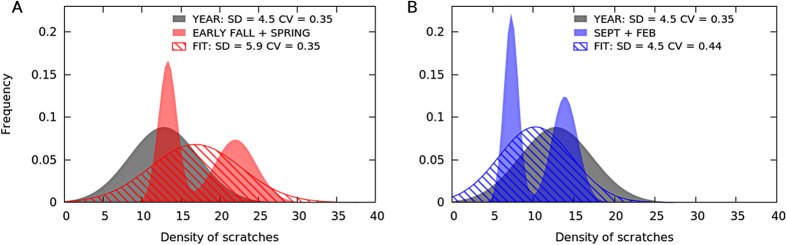
Hypothetical scenarios illustrating how two short events concentrated in separated seasons (“EARLY FALL + SPRING” and “SEPT + FEB” in red and blue, respectively) can only be reliably differentiated from a homogeneous distribution of deaths over the whole year. (“YEAR”, in grey) when considering both SD and CV variability measures. FIT corresponds to the Gaussian fitting implicitly applied when calculating the SD and CV of red and blue bipartite distributions. Panel (**A**) CV does not distinguish two concrete events occurring in spring and early fall, from a homogeneous distribution of deaths over the year. On the contrary, the SD value is different for the two scenarios. Panel (**B)** Opposite to example in Panel (**A**). In this case, only the CV value makes it possible to distinguish two short events concentrated in February and September from deaths evenly distributed through the year.

This complementarity between SD and CV suggests that better classification accuracy could be achieved through a 2D mapping combining both variability measures. Moreover, such a bi-dimensional approach could differentiate a third sort of scenario (i.e. besides single seasonal and longer death events), namely two short events occurring in non-consecutive, different seasons (i.e. spring-autumn and winter-summer, independently of the actual year). In many cases, datasets corresponding to this sort of scenario present the same SD or CV as an equivalent longer death event, but not both variability measures simultaneously. Two illustrative examples are provided in Fig. 4.

### Classification applying a 2D approach combining both variability measures

**Figure 5:**
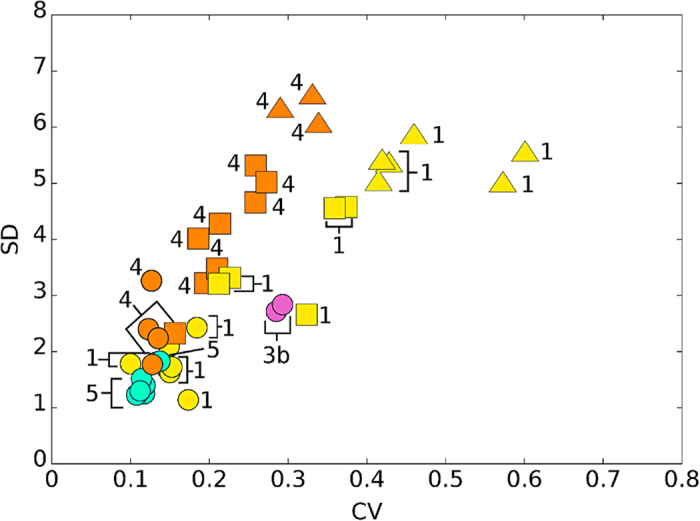
Coefficient of Variation versus Standard Deviation for the training set. Circles represent seasonal or shorter events; squares stand for longer events; triangles correspond to events separated in time. Datapoints correspond to sub-samples of dataset (#1) (*Rangifer tarandus* (Qamanirjuaq, Canada), yellow); dataset (#3b) (*Antilocapra americana* (pronghorn) from Lamont and Rawlins (Wyoming, USA)); dataset (#4) (*Odocoileus hemionus* (Cache La Poudre River, Colorado, USA), orange); dataset (#5) (*Cervus elaphus* (Isle of Rum, Scotland), blue).

In order to test the utility of combining SD and CV measures, we extracted (from the largest modern datasets) sub-samples corresponding to each one of the three scenarios described above, and calculated SD and CV for all of them. Figure 5 shows the obtained values in a bidimensional map, where scenarios are identified by symbols (see the figure’s caption for details).

By visual inspection of Fig. 5, one can easily appreciate how samples appear in three groups corresponding to the three scenarios mentioned above, which are located in three different regions of the SD-CV plane:

- Season-long or shorter time windows (bottom-left region in the figure)
- Longer than a season (central area)
- Separated events that occurred in different non-contiguous seasons (upper-right area)

Once the idea of adopting a bidimensional approach was validated, we needed to divide the whole parameter space (defined by the SD and CV values) into three regions, each one corresponding to one of the categories. This way, any new sample could be classified by positioning it on the 2D plane (using its SD and CV values as coordinates) and checking in which one of the three regions it appeared.

**Figure 6:**
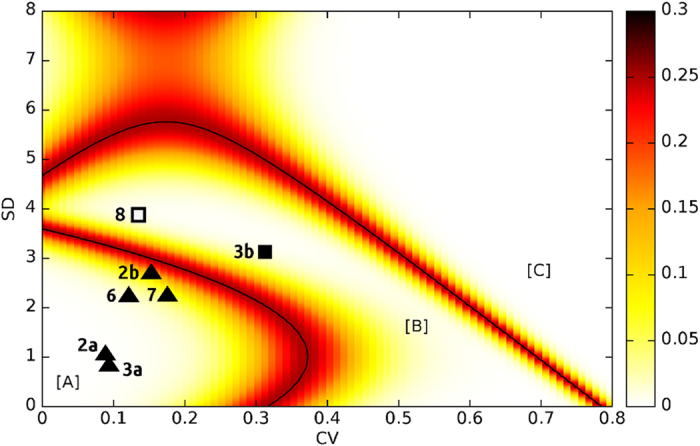
Boundary lines of the three regions with the error probability (heat map). Points correspond to the test dataset: triangles stand for seasonal or shorter events; the black square is an event of deaths distributed over a year; the white square is an unknown case. All the triangles are located in region A, while the squares are in region B and no point falls in region C. Labels refer to the samples ID (Table 1).

Taking this observation as a starting point, we have developed a classification methodology that estimates the boundaries between the three regions based on the naïve Bayes classifier^37^. Details on the methodology are provided in the *Methods* section. The resulting division of the SD-CV plane into three regions is shown in Fig. 6. This plane division allows for the classification of any new case as a function of its position in the SD-CV plane.

The tool also provides a (visual) way to easily assess the accuracy of such a classification. Real cases do not always fit perfectly in the (necessarily simplified) picture encompassed by the three scenarios. This happens, for instance, in the case of two or more short separated events that took place in consecutive seasons or any kind of uneven distribution of deaths through several months. These intermediate situations are likely to be classified both as continuously distributed deaths that occurred all year round (scenario B) or as two separated events that occurred in non-consecutive seasons (scenario C). However, in general, these types of cases will occupy positions close to region boundaries within the SD-CV plane. The closer to a boundary a particular case is, the less clear is its affiliation to any of the two corresponding neighbouring regions separated by such a boundary. More concretely, coloured bands along the boundaries in Fig. 6 indicate the probability that a case located in a particular region did not completely belong to it.

In order to test the reliability of our classification tool, we applied it to the modern samples that we did not include before. Specifically, we mapped 5 datasets (#2a, 2b, 3a, 6, and 7) of seasonal or shorter time spans (region A), one dataset (#3b) of events distributed over one year (region B), and another one whose classification was not clear (#8). As shown in Fig. 5, all the datasets whose classification was known were properly classified. The last one (whose classification was ambiguous) is located in region B, but close to the border with region A. This corresponds to a realistic scenario.

### Using our 2D map approach to classify archaeological assemblages

Notice that the proposed classifier does not distinguish between years of death. Season is the only relevant aspect. This makes the proposed classification method especially suitable for the disambiguation of fossil mortality events, since the resolution of the corresponding archaeological record is of the order of centuries to millennia.

**Figure 7:**
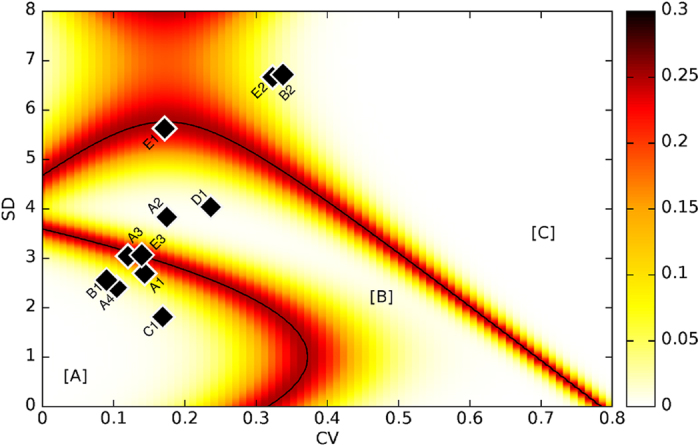
Boundary lines of the three regions with the error probability (heat map) and the fossil samples. (**A1**) *Rangifer tarandus* from Portel-Ouest; (**A2**) *Equus ferus* from Portel-Ouest; (**A3**) *Cervus elaphus* from Portel-Ouest; (**A4**) *Bos/Bison* from Portel-Ouest; (**B1**) *Cervus elaphus* from Abric Romaní level K; (**B2**) *Cervus elaphus* from Abric Romaní level M; (**C1**) *Rangifer tarandus* from Salzgitter Lebenstedt; (**D1**) *Bison priscus* from Taubach; (**E1**) *Equus ferus* from Caune de l’Arago level G; (**E2**) *Cervus elaphus* from Caune de l’Arago level J; (**E3**) *Rangifer tarandus* from Caune de l’Arago level L.

Finally, we also applied the tool to all the archaeological datasets to contrast the expected classification of each case with its positioning in the SD-CV plane. The results, presented in Fig. 7, show a significant agreement with previous interpretations of the archaeological record.

The three samples from the Caune de l’Arago site (E1, E2 and E3) fall in different areas of the SD-CV plane. For level L (E3), the microwear pattern suggests short-term occupation, which fits with the interpretation of short stopovers of reindeer hunters as proposed by de Lumley *et al.*^38^. Remains of red deer from Level J (E2) are related to separated short events, which is consistent with the interpretation of this level as temporary seasonal occupations de Lumley *et al.*^38^. Finally, the sample of horses from level G (E1) falls on the boundary between the regions corresponding to long events and several separated short events. It is thus not possible to differentiate if this level (G) corresponds to a long-term and continuous occupation or to the recurrence of occupations through the year. This uncertainty can be explained from the context of accumulation of the archaeological record. Specifically, level G is a thick (ca. 80 cm) palimpsest (i.e., a sequence of depositional episodes in which successive layers are superimposed on preceding ones), and the distribution of species changes vertically through the level^38^,^39^.

Similarly, the various samples from Portel-Ouest (A1–A4) fall in two areas of the SD-CV plot. Interestingly, these samples correspond to four species from the same level. The accumulation of the reindeer (A1), the red deer (A3), and the large bovid (A4) correspond to seasonal (or shorter) events while the horse (A2) corresponds to a longer continued event. This differential pattern among species can be easily related to known human hunting strategies for different prey at different moments of the year.

At Salzgitter Lebenstedt (C1), the accumulation of reindeer remains corresponds to seasonal (or shorter) events which is consistent with zooarchaeological results indicating autumn hunting of reindeer^40^ (Gaudzinski and Roebroeks, 2000).

Finally, the remains of bison from Taubach (D1) follow a long-term pattern of accumulation which contradict previous results by Moncel and Rivals^41^ who characterized that accumulation as the result of a short-term occupation on the basis of the CV value only. Such results highlight the importance of combining SD and CV to properly estimate the duration of the events.

## Discussion

Summarizing, we used modern data on well-known mass mortality events to develop a tool capable of easily assessing the duration of mortality events from microwear data obtained from the fossil record. Specifically, the tool indicates (with a certain probability value) to what extend a case to be classified matches each one of the following three categories: short (a season or less) events; long-continued event; and two separated short events.

The classification methodology is based on the quantification of the two sources of variability affecting values of scratch density in a sample: heterogeneity due to differences among individuals, and variability resulting from seasonal conditions (e.g. periodic changes in the vegetation). Usual variability measures (i.e. standard deviation and coefficient of variation), calculated separately, cannot properly differentiate short and long mortality events. On the contrary, by bundling them together, the three above-mentioned scenarios can be distinguished.

By comparing the variability values (SD and CV) among the 11 populations, it became evident that variability is independent of the species considered. Samples group depending on the duration of the mortality event. Consequently, the range of CV and SD values considered in our 2D classifier can be used to frame all species of ungulates in seasonal environments.

Specifically, we have divided the space of parameters defined by SD and CV variables into three regions (each of them corresponding to one of the above-mentioned categories) through a naïve Bayesian classifier. We split the modern samples of mass mortality events (for which we knew the exact time window) into two sets. The first one was used to train the classifier, and the resulting regions were checked using the second set. The agreement was perfect. Finally, the tool was applied to the archaeological mortality samples, for which we had theoretical time windows, obtained from other archaeological evidence. All the hypotheses were confirmed by the classification tool. Notice that, despite its power as a simple classifier for ‘archaeological mortality events’, the proposed tools might need to be complemented by a deeper statistical test. This will particularly be the case when sample coordinates (in terms of SD and CV values) locate the sample on a boundary. However, even in those cases, the tool provides a first, highly useful look at the nature of the mortality event.

Finally, the Bayesian nature of our classification tool should recursively allow us to use already classified cases to improve the tool’s accuracy, by informing a priori probabilities. Future efforts will be devoted to explore this possibility.

## Methods

### Datasets

#### Extant samples

After a search for large samples of extant ungulate collections with good records of the date and conditions of death, we collected specimens and established a microwear dataset made of 10 populations (Table 1). Teeth were sampled in each collection and all the moulds and casts produced are now stored in the microwear collection at the Institut Català de Paleoecologia Humana i Evolució Social (Tarragona, Spain). A more comprehensive description of each sample is available in the Supplementary Information.

#### Fossil samples

Fossil samples were selected from archaeological localities ranging in age from the middle Pleistocene (Marine Isotope Stages 14 to 12) and the late Pleistocene (MIS 5e to 3). Summary information of each sample is presented in Table 2. A more comprehensive summary is available in the Supplementary Information.

#### Microwear analysis

Microwear features of dental enamel were examined using a stereomicroscope on high-resolution epoxy casts of teeth following the cleansing, moulding, casting, and examination protocol developed by Solounias and Semprebon^14^ and Semprebon *et al.*^36^. In short, the occlusal surface of the upper or lower second molar of each individual was cleaned using acetone and then 96% alcohol. The surface was moulded using high-resolution silicone (vinylpolysiloxane) and casts were created using clear epoxy resin. All specimens moulded were carefully screened under the stereomicroscope. Those with badly preserved enamel or taphonomic defects (features with unusual morphology and size, or fresh features made during the collecting process or during storage) were removed from the analysis, following King *et al.*^42^.

Casts were observed under incident light with a Zeiss Stemi 2000C stereomicroscope at 35× magnification, using the refractive properties of the transparent cast to reveal microfeatures on the enamel. To avoid inter-observer error, the analysis of microwear was performed by the same person for all samples (FR). We used the classification of features defined by Solounias and Semprebon^14^ and Semprebon *et al.*^36^ and quantified all categories of microwear features. However in this paper, we only present the results on the number of scratches because scratches are related to seasonal changes in diet^14^,^43^. Scratches are elongated microfeatures that are not merely longer than they are wide, but have straight, parallel sides. Scratches were quantified on the paracone of the M2 or the protoconid of the m2 in a standard square area of 0.16 mm^2^ using an ocular reticule. Two areas were analysed on each tooth and the results were averaged to be included in the dataset.

### Determining region boundaries in the 2D SD-CV map using the Naive Bayes classifier

We consider three separated classes of data points corresponding to each one of the considered scenarios: (A) from one month to a season; (B) from four months to a year; (C) events occurred in non-consecutive seasons. Our goal was to determine the boundaries between the regions of the parameters’ space (CV-SD) corresponding to each one of these.

Naive Bayes classifiers are a family of simple probabilistic classifiers based on applying Bayes’ theorem with strong (naive) independence assumptions between the features (in our case, CV and SD). An advantage of naive Bayes is that it only requires a small amount of training data to estimate the parameters necessary for classification. Moreover, since we assume CV and SD to be independent, for each class only their means and variances (and not the entire covariance matrix) need to be determined.

For the construction of the training set, in order to hold a complete control over the time distribution of the events, we selected the modern datasets where the precise date of each death is known (i.e. #1, 4, and 5). We extracted from these datasets sub-samples of data such that their time distribution corresponded to one of the described scenarios with no ambiguity. As a rule of thumb, we fixed the minimum size for a sub-sample to be statistically acceptable equal to fifteen individuals. For smaller sets, we noticed that the SD values have a significant (negative) correlation with the size of the set, hence we discarded them.

For the first two scenarios, we took into account groups of events evenly distributed, avoiding long blank periods or high concentrations of deaths in short time spans. Since none of the three considered datasets includes deaths dated in all the months of the year, we had to relax the requirements in a few cases to obtain a more balanced training set (see Supplementary Information-*Table of the dataset sub-samples included in the training set*). We discarded the sub-sample corresponding to the summer dates in dataset #4 because it was too small. On the contrary, we included three large-enough sub-samples extracted from dataset #3b despite the fact that its time resolution is lower than the general one (i.e. we know the month of the death, but not always the day).

As for the third scenario, we built each sub-sample as two groups of data corresponding to deaths that occurred in two opposite seasons. However, seasons are hardly equally represented within the same dataset. For instance, in the largest one (#1), winter is completely missing, while in #4, summer is under-represented. Consequently, available sub-samples for this scenario are scarce. Hence, we decided to assemble new ones by replicating data corresponding to one of the two considered seasons, thus altering their relative frequencies. In this way, we have been able to obtain up to nine sub-samples with different proportions of events in the cold and in the warm season, theoretically covering all the spectrum of possibilities within the definition of the third scenario (see Supplementary Information–*Table of the dataset sub-samples included in the training set* for further details).

Assuming that the values associated with each class are normally distributed, we associate to each scenario a bidimensional Gaussian distribution. Its parameters are determined as the mean and the standard deviation of the *x* and the *y* values, being *x* and *y* coordinates in the parameter space we want to divide into regions (i.e. SD and CV, respectively, in our case).


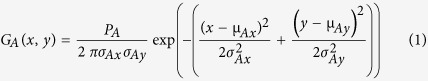



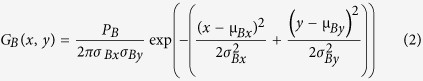



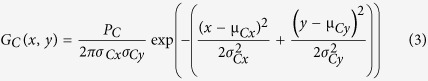


In principle, each distribution should be weighted with a *prior probability* given by the fraction of cases belonging to the corresponding class. In our case, since there is no *a priori* information about real (fossils) case frequencies at hand, we gave each distribution the same weight *P*_*A*_ = *P*_*B*_ = *P*_*C*_ = *p* = *1*/*3*.

Since there is almost no overlap between distribution *G*_*A*_ and distribution *G*_*C*_, we looked for the boundary between class *A* and class *B* and then between class *B* and class *C* as separated situations. In this way, we had two binary classification problems (class 0 *vs.* class 1) and the boundaries could be determined by equating the corresponding distribution functions:


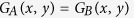
 and 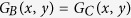, respectively.

Thus, we are dividing the plane into three regions such that the distribution taking the larger value is *G*_*A*_ in region A, *G*_*B*_ in region B, and *G*_*C*_ in region C. The class of any new data point can be therefore easily determined from its position in the parameter space.

The uncertainty of the classification, represented in the heat map of Figs 6 and 7, can be estimated from the variance of the classifier. Its expression is


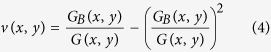


where 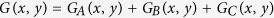 is the total probability distribution for a new mortality event to be found at position 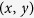 in the SD-CV plane.

**Figure 8:**
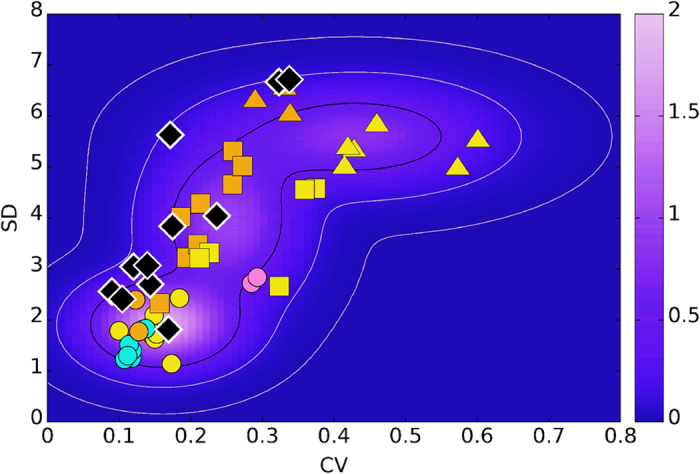
Isolines of G(x,y), the total probability distribution for a mortality event to be found at position (*x, y*) in the SD-CV plane, corresponding to, from the most internal one, a total probability equal to 68%, 95%, 99.7%. Datapoints correspond both to the extant samples (triangles, squares, circles) and the fossil ones (diamonds).

We computed three contour lines of 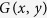 corresponding to a total probability of 68%, 95%, and 99.5%, respectively, of finding a datapoint within the region of the SD-CV plane delimitated by such curves. Notice that all the fossil datasets fall inside the second curve (See Fig. 8). There is no difference between extant and fossil samples in terms of range of SD and CV values and this is precisely, from a statistical point of view, what allows us to use the former to develop a classifier for the latter.

The expected total error is given by the sum of the integrals of each distribution outside the region of the corresponding class (beyond the boundary)


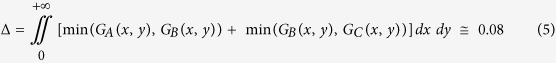


being the overlap of *G*_*A*_ and *Gc* negligible.

## Additional Information

**How to cite this article**: Rivals, F. *et al.* A tool for determining duration of mortality events in archaeological assemblages using extant ungulate microwear. *Sci. Rep.* **5**, 17330; doi: 10.1038/srep17330 (2015).

## Supplementary Material

Supplementary Information

## Figures and Tables

**Figure 1:**
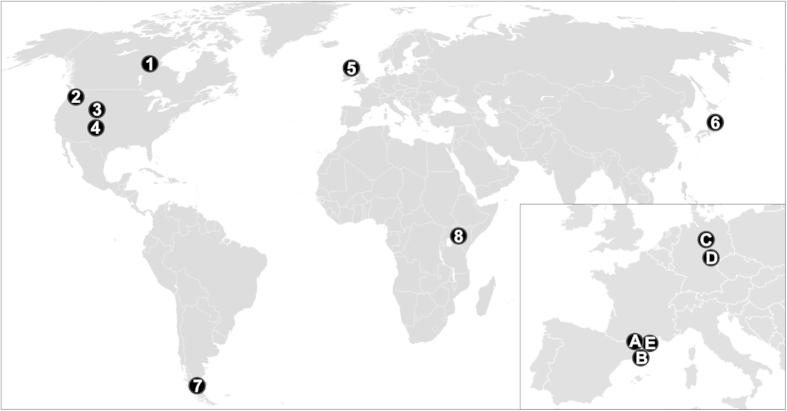
Map of location of the extant and archaeological samples. **Extant samples**: (1) *Rangifer tarandus* (barren ground caribou) from the Qamanirjuaq population (Canada), (2) *Cervus elaphus* (elk) from Mount Saint Helens, Pierce County, and Lewis County (Washington, USA), (3) *Antilocapra americana* (pronghorn) from Green River, Lamont and Rawlins (Wyoming, USA), (4) *Odocoileus hemionus* (mule deer) from Cache La Poudre River (Colorado, USA), (5) *Cervus elaphus* (red deer) from the Isle of Rum (Scotland, UK), (6) *Cervus nippon* (sika deer) from Kinkazan Island (Japan), (7) *Lama guanicoe* (guanaco) from Cardiel lake (Patagonia, Argentina) and (8) *Equus quagga* (plains zebra) from Mount Rumuruti (Kenya). **Archaeological samples**: (**A**) Portel-Ouest (France), (**B**) Abric Romaní (Spain), (**C**) Salzgitter Lebenstedt (Germany), (**D**) Taubach (Germany), (**E**) Caune de l’Arago (France). Source: own elaboration from maps available on Wikimedia Commons free media repository: BlankMap-World6.png (http://commons.wikimedia.org/wiki/File:BlankMap-World6.png), released into the public domain by the authors, and Blank_map_of_Europe.png (http://commons.wikimedia.org/wiki/File:Blank_map_of_Europe.png), licensed under the Attribution-ShareAlike 3.0 Unported license. The license terms can be found on the following link: https://creativecommons.org/licenses/by-sa/3.0/.

**Table 1:** Origin and duration of the mortality events in the samples of extant ungulates.

#	Species	Locality	Cause of death	Duration	N	SD	CV
1	*Rangifer tarandus* (barren ground caribou)	Qamanirjuaq population (Canada)	Shot for population control (3/ 1966 - 7/1968)	years	473	4.555	0.359
2a	*Cervus elaphus* (elk)	Mount Saint Helens (Washington, USA)	Volcanic eruption 05/18/1980	1 day	63	1.054	0.089
2b	*Cervus elaphus* (elk)	Pierce and Lewis Counties (Washington, USA)	Natural deaths(1974 -1978)	years	13	2.800	0.159
3a	*Antilocapra americana* (pronghorn)	Green River (Wyoming, USA)	Fall from a cliff 11/3/1991	1 day	89	0.823	0.094
3b	*Antilocapra americana* (pronghorn)	Lamont and Rawlins Counties (Wyoming, USA)	Natural deaths (1969 - 1972)	years	71	3.152	0.314
4	*Odocoileus hemionus* (mule deer)	Cache La Poudre River (Colorado, USA)	Shot from 04/1961 to 04/1965	years	49	4.706	0.261
5	*Cervus elaphus* (red deer)	Isle of Rum (Scotland, UK)	Winter mortality (1979-2011)	season	174	1.843	0.155
6	*Cervus nippon* (sika deer)	Kinkazan Island (Japan)	Spring mortality in 1984	season	69	2.242	0.122
7	*Lama guanicoe* (guanaco)	Cardiel lake (Patagonia, Argentina)	Winter mortality in 2000	season	91	2.246	0.176
8	*Equus quagga* (plains zebra)	West of Mount Rumuruti (Kenya)	Single breeding population	nd	28	3.952	0.137

# = sample ID, N = sample size (number of specimens analysed), SD = standard deviation, CV = coefficient of variation, nd = no data available.

**Table 2:** Archaeological localities selected to test the tool.

Locality	Age (BP)	Levels	#	Species sampled	N	SD	CV
Portel-Ouest	ca. 45 ka	level F	A1	*Rangifer tarandus*	30	3.097	0.122
			A2	*Equus ferus*	27	3.910	0.178
			A3	*Cervus elaphus*	21	2.764	0.147
			A4	*Bos/Bison*	25	2.459	0.107
Abric Romaní	50.4 ± 0.5 to	level K	B1	*Cervus elaphus*	14	6.970	0.350
	54.5 ± 1.6 ka	level M	B2	*Cervus elaphus*	19	2.629	0.092
Salzgitter Lebenstedt	ca. 58-54 ka	–	C1	*Rangifer tarandus*	81	1.827	0.170
Taubach	116 ± 19 ka	–	D1	*Bison priscus*	22	4.134	0.242
Caune de l’Arago	438 ± 31 ka	level G	E1	*Equus ferus*	38	5.705	0.174
	ca. 500 ka	level J	E2	*Cervus elaphus*	27	6.795	0.329
	ca. 550 ka	level L	E3	*Rangifer tarandus*	30	3.121	0.142

Description of the levels and species sampled. # = sample ID, N = sample size (number of specimens analysed), SD = standard deviation, CV = coefficient of variation.
