# Supplementary material for: A tool for determining duration of mortality events in archaeological assemblages using extant ungulate microwear
Source: Sci Rep. 2015 Nov 30;5:17330. doi: 10.1038/srep17330 (PMC4663483; doi:10.1038/srep17330)
Supplement: Supplementary Information [file srep17330-s1.pdf]

Supplementary Information

A tool for determining duration of mortality events in archaeological assemblages using extant ungulate microwear

Florent Rivals, Luce Prignano, Gina M. Semprebon & Sergi Lozano

SCIENTIFIC REPORTS | 5:17330 | DOI: 10.1038/srep17330

Description of the extant and fossil samples

All datasets used in this paper are available from the Dryad Digital Repository (http://dx.doi.org/10.5061/dryad.9fp8k)

1. Extant samples

Sample #1. Barren ground caribou (*Rangifer tarandus*) from the Qamanirjuaq population (Canada). The Qamanirjuaq population ranges over the southeastern District of Keewatin, in the Northwest Territories, and northern Manitoba and northeastern Saskatchewan. Caribou from the Qamanirjuaq population were killed across all seasons and collected by Canadian Wildlife Service biologists from March 1966 to July 1968, except during the winter months i.e. January to March[1-3]. The collection used in this study consists of skulls and left mandibles of a thousand individuals of all age classes, and both sexes. Sex and age of the individual specimens are known – all caribou specimens had been tagged early in the calving season allowing us to attribute an age at death[2, 4]. The season of death was recorded[2] and the stomach contents were preserved and analyzed for 545 individuals[4]. Dentition was examined for age and sex determination[2, 5], for skeletochronology study[6], for studying the relationship between dental crown height and age[7], for stable isotopes[8,

[9], and for tooth mesowear analysis[10]. The collection we sampled is curated at the Canadian Museum of Nature in Ottawa (Canada).

Sample #2a. Elk (*Cervus elaphus*) killed on May 18th 1980 during the eruption of Mount Saint Helens (USA). The eruption of Mount Saint Helens, a strato-volcano in south-central Washington, resulted in the death of many cervids (including *Cervus elaphus* and *Odocoileus hemionus*) living within a 15 km distance to the north of the volcano[11, 12]. Animals living in the blast zone died from suffocation, explosive shock, traumatic shock, falling timber, or falling pumice blocks resulting from the eruption[11, 13]. Many remains of these cervids were collected in the late summer and early autumn of 1981. The collection is curated at the Burke Museum in Seattle, Washington (USA). The sample was used by Lyman[11-13] to study bone breakage as well as mortality profiles.

Sample #2b. Elk (*Cervus elaphus*) from Lewis and Pierce counties (Washington State, USA) randomly selected from the collection curated at the Burke Museum in Seattle (USA). Animals collected at various seasons from 1974 to 1978.

Sample #3a. Pronghorn (*Antilocapra americana*) that accidentally died on November 3rd, 1991 falling over a cliff in Wyoming (USA). The site, known as Green River Pronghorn Jump, is located in the Reiser Canyon, near the city of Green River (Sweetwater County) in Wyoming[14]. On November 3rd, 1991, a group of 150 pronghorn went over the cliff, hit a small ledge below it, and ended on the talus slope at the base of the cliff. Most individuals perished on that day, except for a few wounded animals that were shot down the following day by the Wyoming Game and Fish Department. Biologists from the latter institution and archaeologists from the Western Wyoming College quantified the animals,

recorded sex and estimated age of the individuals. The site was left undisturbed and was used for various studies which examined various issues in ageing methods, mortality profiles, seasonality, and the taphonomic history of bonebed formation[14, 15]. Mandibles were collected by Patrick Lubinski and Megan Partlow in September 1993, and later by Patrick Lubinski in June 1995 and 1999. A total of 256 mandibles were recovered (out of the 300 that would be originally accumulated). The sample was used in a study to test whether stable isotopes could distinguish assemblages originating as accumulations of individuals derived from multiple populations from mass kills of individuals from a single population[16]. The sample is available at the Zooarchaeology Laboratory at Wyoming University, Laramie (USA).

Sample #3b. Pronghorn (*Antilocapra americana*) representing a 1 year sample from a single population in Wyoming (USA). The pronghorn population is located between Lamont and Rawlins in Carbon County in southern Wyoming[15]. The sample is composed of animals that died at different times of the year between 1969 and 1972. The sample is available at the Zooarchaeology Laboratory at Wyoming University, Laramie (USA).

Sample #4. Rocky Mountain mule deer (*Odocoileus hemionus hemionus*) from Colorado (USA). We sampled a total of 56 individuals from the area around the Cache La Poudre River on the eastern slope of the Front Range in north central Colorado. Mule deer were shot by scientists from the Colorado Division of Wildlife at weekly intervals from April 1961 to April 1965. The sample was previously used for age estimation from incisors[17], estimations of age, sex, weight and season[18] and stable carbon isotope analysis[19]. The material is currently stored at the Department of Archaeology at the University of Calgary (Canada).

Sample #5. Red deer (*Cervus elaphus*) from the Isle of Rum (Scotland). Skulls of naturally dead red deer are collected each year by a research team from the University of Edinburgh on the Isle of Rum[20-22]. We sampled the winter mortality specimens from the skull collection on the island that died from 1979 to 2011. All data were collected on red deer from the North Block of the island, a 12 km^2 study area. The deer population is monitored on an individual basis since the early 1970s. Censuses are conducted on a regular basis to control the population. Mortality searches are conducted through the mortality period (January to April) to locate carcasses of missing animals that might have died. Date of death is determined depending on the weekly monitoring of the study area. Deer carcasses are identified and the jaw of all individuals are removed, labeled and stored. The material is stored at the research station of the University of Edinburgh on the Isle of Rum. The bone collection has been used for analysis of tooth wear[23], estimation of age[24-26], stable isotopes[20, 21].

Sample #6. Sika deer (*Cervus nippon*) from Kinkazan Island (Japan) died from late winter to early spring mortality. Kinkazan Island is 9.6 km^2 in size and located in northern Japan, 600 m off the Oshika Peninsula, facing the Pacific Ocean. Because of religious reasons, the island has been conserved, and sika deer are protected. However, a mass mortality happened in the spring of 1984 after a record-breaking snowy winter[27, 28]. Seiki Takatsuki and colleagues located about 300 carcasses. When carcasses were found, the skulls were collected. The skulls were cleaned and the sampling was performed at Azabu University. The samples were used in various studies to analyze tooth mesowear[29], tooth wear[30], relation between feeding and morphology[31] and to investigate the relation

between tooth microwear and bamboo feeding[32]. All specimens belong to the collections of the University Museum of the University of Tokyo (UMUT), Japan.

Sample #7. Guanaco (*Lama guanicoe*) from Patagonia (Argentina) dead during a single event in winter 2000. The sample analyzed comes from an area at the West of the Cardiel Lake in the area called "Cañadones" in Southern Patagonia (Province of Santa Cruz). Six faunal assemblages were surveyed: four under rockshelters (or *aleros*) and two in open air sites. Arturo Olivero, employee at "La Carlina" farm, knew those guanaco assemblages and pointed out that they were formed during the intense snowfalls of 2000[33, 34]. A total of 100 individuals were sampled: 77 skulls collected by Diego Rindel and Juan Bautista Belardi in 2009 and 2010 and curated at the *Instituto Nacional de Antropologia y Pensamiento Latinoamericano* (INAPL) and 23 individuals that were still *in situ* in the Cardiel Lake area and were directly sampled during a field trip in November 2011 (after sampling the skulls were left *in situ*)[35].

Sample #8. Plains zebra (*Equus quagga*) from Kenya. The sample comes from a single locality at Rumuruti (west of Mount Kenya) and from a single breeding population. The locality is at an altitude around 1770 m. No data is available about the context of collection of the specimens. The collection of skulls was given to the Vertebrate Paleontology Laboratory at University of Texas (Austin) in 1961 by L.S.B. Leakey.

2. Fossil samples

Fossil samples were selected from archaeological sites in Europe. Selection criteria included:

Sample A. Portel-Ouest (France)

The Portel-Ouest cave (Loubens, France) lies at an altitude of 485 m above sea level on the northern slope of the Pyrenees. It has been excavated by Joseph and Jean Vézian and yielded Mousterian tools[36] and abundant fossil mammal material[37]. The deposits correlate with MIS 5 to 2 but material was sampled only from level F which is the oldest Mousterian level, and is dated to about 45 ka (MIS 3)[38]. It produced the most important archaeological sample from Portel-Ouest including faunal and Neanderthal remains, stone tools and other implements. We sampled the following species: *Rangifer tarandus*, *Equus ferus*, *Cervus elaphus*, and a large bovid (*Bos* or *Bison*). The material we analysed is stored at the Centre Européen de Recherches Préhistoriques in Tautavel (France).

Sample B. Abric Romaní (Spain)

The Abric Romaní is located in Capellades (Catalonia, Spain), at 50 km from Barcelona. The shelter is today at 317 m above sea level. The stratigraphic sequence is about 20 m thick and 27 archeological levels were identified. This sequence was dated by U-Series to 40-70 ka BP[39, 40]. Two levels (K and M), all belonging to MIS 3 and all being recently excavated where selected. Level M is dated between 54.5±1.6 and 52.2±1.6 ka BP, and level K between 52.3±0.6 and 50.4±0.5 ka BP[39]. We used data on the red deer remains

from these two levels[41]. The material analysed is currently stored at the Institut Català de Paleoecologia Humana i Evolució Social (Spain).

Sample C. Salzgitter Lebensted (Germany)

The site of Salzgitter Lebenstedt is located approximately 50 km south-east of the town of Hannover (Germany). The site exposes a Pleistocene channel filled with Weichselian fluvial sediments including interposed still-water deposits such as fine sand, mud and peat[42]. The fluvial sediments are about 2 m thick and can be separated into three subunits. The archaeological material was mainly distributed in the deposits of the intermediate unit 2 and the lower unit 1. On the basis of pollen and sedimentological evidence, it was suggested that the site dates to the Oerel Interstadial, dated by C14 around 58-54 ka BP[43], i.e. the beginning of MIS 3. Remains left by Neanderthals indicate a specific hunting activity on *R. tarandus*[44, 45]. The material is stored at the Braunschweigisches Landesmuseum at Wolfenbüttel (Germany).

Sample D. Taubach (Germany)

The Taubach sequence is part of a complex of travertine exposed along the slopes of the Ilm river valley close to the town of Weimar (Germany). The sequence contains a homogeneous archaeological horizon represented by a sandy travertine, the 'Knochensand'[46-48]. Flint artefacts and mammal remains were recovered from this horizon. Analyses of small mammals[49], radiometric dates of 116±19 ka[50], analysis of flint artifacts[51], and the large mammal fauna support the conclusion that the Taubach travertine should be dated into the Eemian interglacial (MIS 5e). The observation of cut marks on bones of most of the species and their unusually high frequencies, combined with the mortality profiles of prey animals, indicates a high degree of anthropogenic involvement

in this accumulation[46]. At least a large part of the faunal assemblage must have resulted from deliberate hunting of large mammals[46]. The material analyzed is housed at the Forschungsstation für Quartärpaläontologie at Weimar, Forschungsinstitut und Naturmuseum Senckenberg (Germany).

Sample E. Caune de l'Arago (Tautavel, France)

The Caune de l'Arago is a large karstic cavity located in the Eastern Pyrenees (France). The cave has been excavated every year since 1964 by teams directed by Prof. Henry de Lumley. The deposits are almost 15 m thick and cover a period of 690,000 to 100,000 years[52]. Most faunal assemblages are associated with abundant stone tools, and human remains are found in some levels. Zooarchaeological analyses combined with data from lithic assemblages and sedimentological studies show that these levels include those of long duration (level G), temporary seasonal accumulations (levels E, F, and J), accumulations related to short hunting stopovers (levels K and L), and bivouac assemblages (level P)[53]. We sampled *Rangifer tarandus* from level L (about 550 ka), *Cervus elaphus* from level J (about 500 ka), *Equus ferus* from level G (438±31 ka)[54]. Material is housed at the Centre Européen de Recherches Préhistoriques in Tautavel, France.

Table of the datasets sub-samples included in the training set.

We report the relevant information about the sub-samples included in the training set: the dataset they are extracted from, the corresponding CV and SD values, range of days (when known) and months of death, and the total number of individuals. If not otherwise indicated, all the deaths in the data range have been included, independently of the year.

In the case of separated events (Region C), beside the total size of the sample, we reported the number of individuals in each dates range, explicitly indicating when they are replicated.

**Region A: event's duration equal or shorten than one season**					
**Dataset**	**CV**	**SD**	**Dates range**	**Size**	**Notes**
**#1**	0.100	1.785	7-22apr	136	N1966=51 - N1967=45 - N1968=40
**#1**	0.147	1.683	15-24sep	59	N1966=13 - N1967=46
**#1**	0.150	1.628	2-17jul	44	1968 only
**#1**	0.173	1.140	21nov-12dec	118	N1966=58 - N1967=58
**#1**	0.184	2.428	25may-17jul	162	N1966=31 - N1967=43 - N1968=88
**#1**	0.152	1.726	23june-21sep	104	Blank period: august
**#1**	0.149	2.091	25may-26jun	118	N1966=31- N1967=43 - N1968=44
**#1**	0.132	1.807	1-26jun	106	N1966=31- N1967=31 - N1968=44
**#5**	0.118	1.252	1-29feb	37	Distributed through several years
**#5**	0.118	1.399	1-31mar	65	“
**#5**	0.114	1.518	2-29apr	34	“
**#5**	0.137	1.830	2apr-23may	43	“
**#5**	0.108	1.228	1-31jul	15	“
**#5**	0.112	1.300	4-31oct	21	“
**#4**	0.128	1.772	2jan-22mar	16	“
**#4**	0.123	2.394	12apr-20jun	15	“

**#3b**	0.293	2.838	jun-jul	20	“
**#3b**	0.285	2.719	jun-ago	28	“
**#3b**	0.293	2.838	sep-oct	22	“

**Region B: events longer than a season**					
**Dataset**	**CV**	**SD**	**Dates range**	**Size**	**Notes**
**#1**	0.324	2.658	15sep-12dec	116	N1966=15-N1967=101
**#1**	0.226	3.315	7apr-24sep	357	N1966=95-N1967=134-N1968=128
**#1**	0.212	3.211	7apr-17jul	128	N1966=82-N1967=88-N1968=128<br>Blank period: 23apr-24may
**#1**	0.359	4.555	7apr-12dec	473	N1966=157-N1967=189-N1968=127
**#1**	0.374	4.577	10apr-12dec	189	1967only<br>Blank periods: 23apr-24may, aug, oct
**#4**	0.210	3.480	2jan-20jun	31	Distributed through several years
**#4**	0.186	4.015	12apr-18sep	23	“
**#4**	0.259	5.316	2jul-26dec	18	“
**#4**	0.157	2.327	22oct-20mar	26	“
**#4**	0.258	4.658	2jan-26dec	49	“
**#4**	0.272	5.019	2jul-30mar	39	“
**#4**	0.195	3.220	fall+20jun	41	“
**#4**	0.214	4.280	12apr+26dec	33	“

**Region C: separated events**								
**Dataset**	**CV**	**SD**	**Dates range event 1**	**Dates range event 2**	**Size**	**Size1**	**Size2**	**Notes**
**#1**	0.415	4.994	7apr	21nov	13	N1=6	N2=7	1966 only
**#1**	0.428	5.322	7apr-20jun	21sep-12dec	311	N1=136	N2=175	
**#1**	0.460	5.823	7apr-22apr	21nov-12dec	252	N1=136	N2=116	
**#1**	0.573	4.968	25-29may	1-12dec	49	N1=12	N2=37	
**#1**	0.419	5.370	9apr	22nov	18	N1=9	N2=9	1966 only

**#1**	0.601	5.515	28may	2dec	17	N1=12	5	1967 only
**#4**	0.339	6.026	2jul-18sep	2jan-20mar	24	N1=8	N2=16	
**#4**	0.290	6.295	4x(2jul-18sep)	2jan-20mar	48	4xN1=32	N2=16	replicated
**#4**	0.331	6.536	2x(2jul-18sep)	2jan-20mar	32	2xN1=16	N2=16	replicated

References

1. Miller F. L. Eruption and attrition of mandibular teeth in barren-ground caribou. *Journal of Wildlife Management* **36**, 606-612 (1972).

2. Miller F. L. Biology of the Kaminuriak population of barren-ground caribou. Part 2: Dentition as an indicator of age and sex; composition and socialization of the population. *Canadian Wildlife Service Report Series* **31**, 1-87 (1974).

3. Parker G. R. Biology of the Kaminuriak population of barren-ground caribou. Part 1: Total numbers, mortality, recruitment, and seasonal distribution. *Canadian Wildlife Service Report Series* **20**, 1-93 (1972).

4. Miller D. R. Biology of the Kaminuriak population of barren-ground caribou. Part 3: Taiga winter range relationships and diet. *Canadian Wildlife Service Report Series* **36**, 1-37 (1976).

5. Morrison D. Estimating the age and sex of caribou from mandibular measurements. *Journal of Archaeological Science* **24**, 1093-1106 (1997).

6. Pike-Tay A. Variability and synchrony of seasonal indicators in dental cementum microstructure of the Kaminuriak caribou population. *Archaeofauna* **4**, 273-284 (1995).

7. Pike-Tay A., Morcomb C. A. & O'Farrell M. Reconsidering the Quadratic Crown Height Method of age estimation for *Rangifer* from archaeological sites. *Archaeozoologia* **11**, 145-174 (2000).

8. Drucker D., Bocherens H., Pike-Tay A. & Mariotti A. Isotopic tracking of seasonal dietary change in dentine collagen: preliminary data from modern caribou. *Comptes Rendus de l'Académie des Sciences, Paris, Sciences de la Terre et des planètes* **333**, 303-309 (2001).

9. Drucker D. G., Hobson K. A., Münzel S. C. & Pike-Tay A. Intra-individual variation in stable carbon ($\delta^{13}C$) and nitrogen ($\delta^{15}N$) isotopes in mandibles of modern caribou of Qamanirjuaq (*Rangifer tarandus groenlandicus*) and Banks Island (*Rangifer tarandus pearyi*): Implications for tracing seasonal and temporal changes in diet. *International Journal of Osteoarchaeology* **22**, 494-504 (2012).

10. Rivals F. & Solounias N. Differences in tooth microwear of populations of caribou (*Rangifer tarandus*, Ruminantia, Mammalia) and implications to ecology, migration, glaciations and dental evolution. *Journal of Mammalian Evolution* **14**, 182-192 (2007).

11. Lyman R. L. Broken bones, bone expediency tools, and bone pseudotools: lessons from the blast zone arounds Mount St. Helens, Whashington. *American Antiquity* **49**, 315-333 (1984).

12. Lyman R. L. Taphonomy of cervids killed by the May 18, 1980, volcanic eruption of Mount St. Helens, Washington, U.S.A. In: *Bone modification* (eds Bonnichsen R., Sorg M. H.) 149-167 (Center for the Study of the First Americans, University of Maine, 1989).

13. Lyman R. L. On the analysis of vertebrate mortality profiles: sample size, mortality type, and hunting pressure. *American Antiquity* **52**, 125-142 (1987).

14. Lubinski P. M. & O'Brien C. J. Observations on seasonality and mortality from a recent catastrophic death assemblage. *Journal of Archaeological Science* **28**, 833-842 (2001).

15. Lubinski P. M. Estimating age and season of death of pronghorn antelope (*Antilocapra americana* Ord) by means of tooth eruption and wear. *International Journal of Osteoarchaeology* **11**, 218-230 (2001).

16. Fenner J. N. The use of stable isotope ratio analysis to distinguish multiple prey kill events from mass kill events. *Journal of Archaeological Science* **35**, 704-716 (2008).

17. Erickson J. A. & Seliger W. G. Efficient sectioning of incisors for estimating ages of mule deer. *Journal of Wildlife Management* **33**, 384-388 (1969).

18. Oetelaar G. A. *Deer remains in archaeology with special reference to the mandible of mature Rocky Mountain mule deer*. Simon Fraser University (1981).

19. Hobson K. A. & Schwarcz H. P. The variation in $\delta^{13}C$ values in bone collagen for two wild herbivore populations: Implications for palaeodiet studies. *Journal of Archaeological Science* **13**, 101-106 (1986).

20. Stevens R. E., Balasse M. & O'Connell T. C. Intra-tooth oxygen isotope variation in a known population of red deer: Implications for past climate and seasonality reconstructions. *Palaeogeography, Palaeoclimatology, Palaeoecology* **301**, 64-74 (2011).

21. Stevens R. E., Lister A. M. & Hedges R. E. M. Predicting diet, trophic level and palaeoecology from bone stable isotope analysis: a comparative study of five red deer populations. *Oecologia* **149**, 12-21 (2006).

22. Virtanen R., Edwards G. R. & Crawley M. J. Red deer management and vegetation on the Isle of Rum. *Journal of Applied Ecology* **39**, 572-583 (2002).

23. Nussey D. H., Metherell B., Moyes K., Donald A., Guinness F. E. & Clutton-Brock T. H. The relationship between tooth wear, habitat quality and late-life reproduction in a wild red deer population. *Journal of Animal Ecology* **76**, 402-412 (2007).

24. Lowe V. P. W. Teeth as indicators of age with special reference to red deer (*Cervus elaphus*) of known age from Rhum. *Journal of Zoology* **152**, 137-153 (1967).

25. Mitchell B. Growth layers in dental cement for determining the age of red deer (*Cervus elaphus* L.). *Journal of Animal Ecology* **36**, 279-293 (1967).

26. Pérez-Barbería F. J., Duff E. I., Brewer M. J. & Guinness F. E. Evaluation of methods to age Scottish red deer: the balance between accuracy and practicality. *Journal of Zoology* **294**, 180-189 (2014).

27. Takatsuki S., Miura S., Suzuki K. & Ito-Sakamoto K. Age structure in mass mortality in the sika deer (*Cervus nippon*) population on Kinkazan Island, Northern Japan. *Journal of the Mammalogical Society of Japan* **15**, 91-98 (1991).

28. Takatsuki S., Suzuki K. & Suzuki I. A mass-mortality of Sika deer on Kinkazan Island, northern Japan. *Ecological Research* **9**, 215-223 (1994).

29. Kubo M. O. & Yamada E. The inter-relationship between dietary and environmental properties and tooth wear: Comparisons of mesowear, molar wear rate, and hypsodonty index of extant sika deer populations. *PLoS ONE* **9**, e90745 (2014).

30. Ozaki M*., et al.* The relationship between food habits, molar wear and life expectancy in wild sika deer populations. *Journal of Zoology* **280**, 202-212 (2010).

31. Ozaki M*., et al.* Correlations between feeding type and mandibular morphology in the sika deer. *Journal of Zoology* **272**, 244-257 (2007).

32. Rivals F., Takatsuki S., Albert R. M. & Macià L. Bamboo feeding and tooth wear of three sika deer (*Cervus nippon*) populations from northern Japan. *Journal of Mammalogy* **95**, 1043-1053 (2014).

33. Belardi J. B. & Rindel D. Taphonomic and archeological aspects of massive mortality processes in guanaco (*Lama guanicoe*) caused by winter stress in Southern Patagonia. *Quaternary International* **180**, 38-51 (2008).

34. Rindel D. & Belardi J. B. Mortandad catastrófica de guanacos por estrés invernal y sus implicaciones arqueológicas: El sitio Alero Los Guanacos 1, Lago Cardiel (Provincia de Santa Cruz, Argentina). *Magallania* **34**, 139-155 (2006).

35. Rivals F., Rindel D. & Belardi J. B. Dietary ecology of extant guanaco (*Lama guanicoe*) from Southern Patagonia: seasonal leaf browsing and its archaeological implications. *Journal of Archaeological Science* **40**, 2971-2980 (2013).

36. Vézian J. Les fouilles à l'entrée du Portel-Ouest (Loubens, Ariège): stratigraphie générale et passage du Paléolithiqe moyen et Paléolithique supérieur. *Bulletin de la Société Préhistorique de l'Ariège* **44**, 225-261 (1989).

37. Gardeisen A. Middle Palaeolithic subsistence in the West Cave of "Le Portel" (Pyrénées, France). *Journal of Archaeological Science* **26**, 1145-1158 (1999).

38. Bahain J.-J. *La méthode de datation par résonance de spin électronique (ESR) au Muséum national d'histoire naturelle. Vingt ans de recherches méthodologiques et d'applications géochronologiques*. Université Michel de Montaigne (2007).

39. Bischoff J. L., Julia R. & Mora R. Uranium-series dating of the Mousterian occupation at Abric Romani, Spain. *Nature* **332**, 68-70 (1988).

40. Bischoff J. L., *et al.* Dating of the basal Aurignacian sandwich at Abric Romani (Catalunya, Spain) by radiocarbon and uranium-series. *Journal of Archaeological Science* **21**, 541-551 (1994).

41. Rivals F., Schulz E. & Kaiser T. M. Late and middle Pleistocene ungulates dietary diversity in Western Europe indicate variations of Neanderthal paleoenvironments through time and space. *Quaternary Science Reviews* **28**, 3388-3400 (2009).

42. Preul F. Die Fundschichten im Klärwerksgelände von Salzgitter-Lebenstedt und ihre Einordnung in die Schichtenfolge des Quartär. In: *Der Altsteinzeitlische Fundplatz Salzgitter-Lebenstedt. Teil II. Naturwissen-schaftliche Untersuchungen* (eds Busch R., Schwabedissen H.) 9-99 (Böhlau, 1991).

43. Behre K.-E. & van der Plicht J. Towards an absolute chronology for the last glacial period Europe: radiocarbon dates Oerel, northern Germany. *Vegetation History and Archaeobotany* **1**, 111-117 (1992).

44. Gaudzinski S. On the variability of Middle Palaeolithic procurement tactics: The case of Salzgitter Lebenstedt, Northern Germany. *International Journal of Osteoarchaeology* **10**, 396-406 (2000).

45. Gaudzinski S. & Roebroeks W. Adults only. Reindeer hunting at the Middle Palaeolithic site Salzgitter Lebenstedt, Northern Germany. *Journal of Human Evolution* **38**, 497-521 (2000).

46. Bratlund B. Anthropogenic factors in the thanatocoenose of the last interglacial travertines at Taubach (Germany). In: *The role of early humans in the accumulation of European Lower and Middle Palaeolithic bone assemblages* (eds Gaudzinski S., Turner E.) 255-262 (Verlag des Römisch-Germanischen Zentralmumeums, 1999).

47. Gaudzinski S. Monospecific or species-dominated faunal assemblages during the Middle Paleolithic in Europe. In: *Transitions before the transition* (eds Hovers E., Kuhn S. L.) 137-147 (Springer, 2006).

48. Bratlund B. Taubach revisited. *Jahrbuch des Römisch-Germanischen Zentralmuseums Mainz* **46**, 61–174 (2000).

49. Heinrich W.-D. Biostratigraphische Aussagen der Säugetierpaläontologie zur Altersstellung pleistozäner Travertinfundstätten in Thüringen. *Berliner Geowissenschaftliche Abhandlungen* **13**, 251-267 (1994).

50. Brunnacker K., Jager K. D., Gend H., Preuss J. & Grün R. Radiometrische Untersuchungen zur Datierung mitteleuropäischer Travertinvorkommen. *Ethnographisch-Archäologische Zeitschrift* **24**, 217-266 (1983).

51. Schäfer D., Heinrich W.-D., Böhme G. & Steiner W. Aspects of the geology, palaeontology and archaeology of the travertine site of Weimar-Ehringsdorf (Thuringia, Central Germany). *Courier Forschungsinstitut Senckenberg* **259**, 141-147 (2007).

52. de Lumley H., Fournier A., Park Y. C., Yokoyama Y. & Demouy A. Stratigraphie du remplissage pléistocène moyen de la Caune de l'Arago à Tautavel. Etude de huit carottages effectués de 1981 à 1983. *L'Anthropologie* **88**, 5-18 (1984).

53. de Lumley H., *et al.* Habitat et mode de vie des chasseurs paléolithiques de la Caune de l'Arago (600 000 - 400 000 ans). *L'Anthropologie* **108**, 159-184 (2004).

54. Falguères C., *et al.* New ESR and U-series dating at Caune de l'Arago, France: A key-site for European Middle Pleistocene. *Quaternary Geochronology*, doi: 10.1016/j.quageo.2015.1002.1006 (in press).
